# COVAX and COVID‐19 Vaccine Inequity: A case study of G‐20 and African Union

**DOI:** 10.1002/puh2.185

**Published:** 2024-05-09

**Authors:** Anjali Pushkaran, Vijay Kumar Chattu, Prakash Narayanan

**Affiliations:** ^1^ Prasanna School of Public Health, Manipal Academy of Higher Education Manipal Karnataka India; ^2^ Department of Health Policy London School of Economics and Political Science London UK; ^3^ Center for Evidence‐based Diplomacy Global Health Research and Innovations (GHRIC) Toronto Ontario Canada; ^4^ Department of OS & OT, Temerty Faculty of Medicine University of Toronto Toronto Ontario Canada; ^5^ United Nations University‐Institute on Comparative Regional Integration Studies (UNU‐CRIS) Bruges Belgium; ^6^ School of Public Health, University of Alberta Edmonton Alberta Canada; ^7^ Department of Health Policy Prasanna School of Public Health, Manipal Academy of Higher Education Manipal Karnataka India

**Keywords:** African Union, COVAX, COVAX alliance, COVID‐19, G20, low‐ and middle‐income countries, vaccine equity

## Abstract

As the world has a history of vaccine nationalism, especially during the 2009 Swine flu pandemic, the COVAX alliance, a globally collaborated mechanism, was created by World Health Organization (WHO), GAVI, and UNICEF to address the inequity of COVID‐19 vaccines. One of the primary aims of this alliance was to deliver vaccines to low‐ and middle‐income countries (LMICs), which otherwise have less or no capacity to access vaccines from the open market. It is crucial to explore the contribution of COVAX in bridging the gap in equity, accessibility, and affordability of COVID‐19 vaccines between high‐ and low‐income countries (LICs). We selected Group 20 (G20) COVAX participants and the African Union (AU) as case studies to estimate these gaps. The bilateral purchase data shows that by December 2021, the G20 countries had vaccines more than double their population, whereas the AU could procure only about one fifth (19%) of their population. Out of 52 AU countries whose data was available, only 21 of them could strike a bilateral deal with vaccine manufacturers. Even after COVAX delivery, the share of the population that could be vaccinated in AU was just 36.8%, less than the target of WHO (40%) for December 2021. It was found that the COVAX alliance worked better than the open market competition for LMICs and LICs. The cost of vaccinating 20% of the population was 0.7% of the current health expenditure for G20 countries, whereas AU countries had to spend 5.5%. COVAX bears more cost (1%–3%) for AU countries than G20 countries (less than 1%). COVAX made COVID‐19 vaccines more affordable and accessible to these countries. However, LICs were disproportionately affected even with the COVAX Facility mechanism owing to their lack of vaccine deployment infrastructure.

## Graphical abstract

COVAX delivery to African Union states was inadequate to achieve the WHO target of 40% coverage along with its own target of 20% by December 2021. However, the alliance was a better alternative for LMICs and LICs than open vaccine market. Moreover, COVAX has made the COVID‐19 vaccines more affordable to AFU than G20.

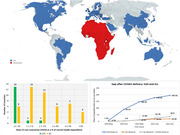

## INTRODUCTION

The COVID‐19 disease exposed alarming fissures in our existing health systems and overwhelmed healthcare facilities worldwide. The global scientific community quickly identified COVID‐19 vaccines as the most vital worldwide intervention to end the pandemic. They were expected to limit the virus circulation and protect against the severity of the illness, hospitalization, and death [1]. The world developed around 40 COVID‐19 vaccines to end the pandemic. At a media briefing on the COVID‐19 pandemic in March 2020, the World Health Organization (WHO) stated [2]:

We are one humanity with one common enemy.

It was correct that the world suffered from COVID‐19, but were all countries united in response? It was evident that all countries were looking forward to the availability of successful COVID‐19 vaccines. Even though efficient vaccines were developed in record time, more pressing questions were to be asked: Which countries would get access to these vaccines from the market? Could the low‐and‐middle‐income countries (LMICs) afford to purchase the vaccines if they were left entirely to a competitive open market?

It was essential to ask these questions as global vaccine inequity is not unknown. Concerns regarding fair access to resources became prominent earlier in 2009 when a new strain of influenza A (H1N1) emerged and spread globally. Affluent nations secured substantial advance orders for the 2009‐H1N1 vaccine, essentially acquiring nearly all the available manufacturing capacity. Developing nations, along with the WHO, raised concerns about the unequal distribution of access to the vaccine by developed countries. The developing countries received donations from developed countries, but it was insufficient to vaccinate their populations, further exacerbating the imbalance in access [3].

The WHO envisioned a global vaccination strategy for COVID‐19 vaccines [4], as the pandemic could easily aggravate the already existing health inequalities [5]. The WHO strategy stressed equity, inclusivity, and accessibility of COVID‐19 vaccines as a response. A globally collaborated mechanism, the COVAX alliance, was launched in April 2020 to perform COVID‐19 vaccine procurement and allocation based on the notion of global vaccine equity.

The COVAX Alliance was a novel approach to international partnership in global health intended to achieve the worthy goal of “COVID‐19 Vaccine Equity.” This alliance followed the public–private partnership model among global health actors, regulatory agencies, pharmaceutical companies, and private philanthropy organizations. The main mechanisms of COVAX—procurement, allocation, and delivery—were meant to work inter‐connectedly, and the framework of COVAX was meant to evolve to the pandemic needs. The initial goal of the alliance was to deliver 2 billion doses by the end of 2021.

About 51% of the world population, that is, 4 billion people, live in LMICs, but their share of fully vaccinated populations was alarmingly low. The Global Dashboard on COVID‐19 Vaccine Equity by UNDP reveals that by December 2021, only 4% of the population were fully vaccinated in low‐income countries (LICs) and 35% for LMICs. In contrast, high‐income countries (HICs) vaccinated 70% of their population [6]. This was far from achieving WHO's goal of vaccinating population coverage of 40% in all countries by December 2021 [7].

### Bilateral deals and COVAX delivery

To ensure access to a vaccine, governments from more affluent nations have entered into direct one‐on‐one agreements with vaccine manufacturers to reserve supplies for their citizens. By December 2021, when 9.72 billion doses were administered worldwide, the LICs received only 73 million doses, whereas HICs and upper‐middle‐income countries cornered 6.25 billion doses [8]. In the initial year of COVID‐19 vaccine distribution, HICs achieved vaccination rates ranging from 75% to 80%, whereas LICs vaccinated less than 10% [9].

**FIGURE 1:**
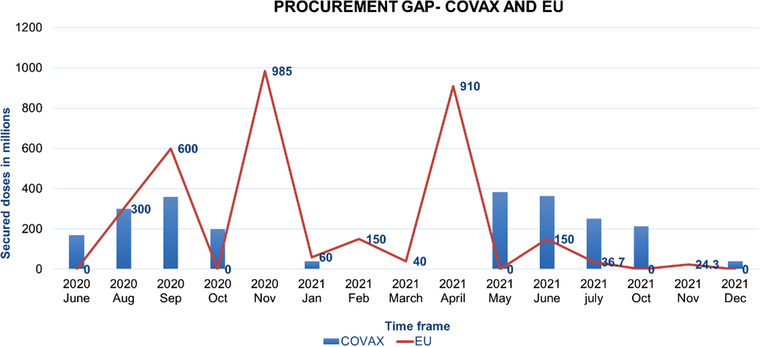
Procurement gap between COVAX and the EU, 2021 [13]. The total purchase deals for the EU and COVAX Facility were taken from the WHO‐IMF COVID‐19 vaccine tracker from June 2020 to December 2021. Both the data are plotted with a time frame on the *x*‐axis and secured doses in millions on the *y*‐axis. The blue vertical bars represent COVAX purchases, and the redline denotes EU purchases in the same time frame.

The actual delivery of COVAX was far less than its supply forecasts. Assessing the framework gaps and implementation challenges of COVAX, it was clear that the piling up of bilateral deals by HICs played the most significant factor in COVAX's failure [10]. The COVAX Facility could deliver only 957 million vaccine doses by the end of 2021, less than 10% of the total globally administered doses, less than half of what was promised initially by the alliance. Moreover, one‐fifth of these doses were reserved for HICs because of contractual obligations by the COVAX Facility [11]. The HICs took advantage of their powerful economic and political positions and secured an unjustifiably large number of doses of vaccines for their population [12]. As a result, COVAX did not get enough supply from the pharma companies, which delayed the vaccine allocations and deliveries to LMICs and LICs. This was evident from the total number of purchased vaccine doses by COVAX and EU from the WHO‐IMF COVID‐19 vaccine tracker [13]. From October 2020 to May 2021, the COVAX procurement was almost nil compared to the EU (Figure 1). The EU procured around 2 billion doses in these 6 months. This was almost equal to the entire target of the COVAX alliance.

### Inequity between G20 and the African continent

The inequity faced by the African continent was high, with only 2.4% of the people having been vaccinated compared to 41% of the North American population and 38% of the European population in 2021. The COVAX Facility has delivered only 83 million vaccine (by June 2021) doses to 75 countries, that is, only 4% of the global supply, and one fifth of this was for HICs [14], and only 11% of all people in LICs have received at least one dose, and a significant gap in accessibility was in the African continent [15]. With publicly available data, we analyzed inequity in COVID‐19 vaccine access of two political groups, the Group 20 (G20) and the African Union (AU). We selected these groups because the G20 represents the HICs with the maximum purchasing power and accessibility to COVID‐19 vaccines, and the AU represents the LICs and LMICs that lack the political and purchasing power to access the vaccines.

**FIGURE 2:**
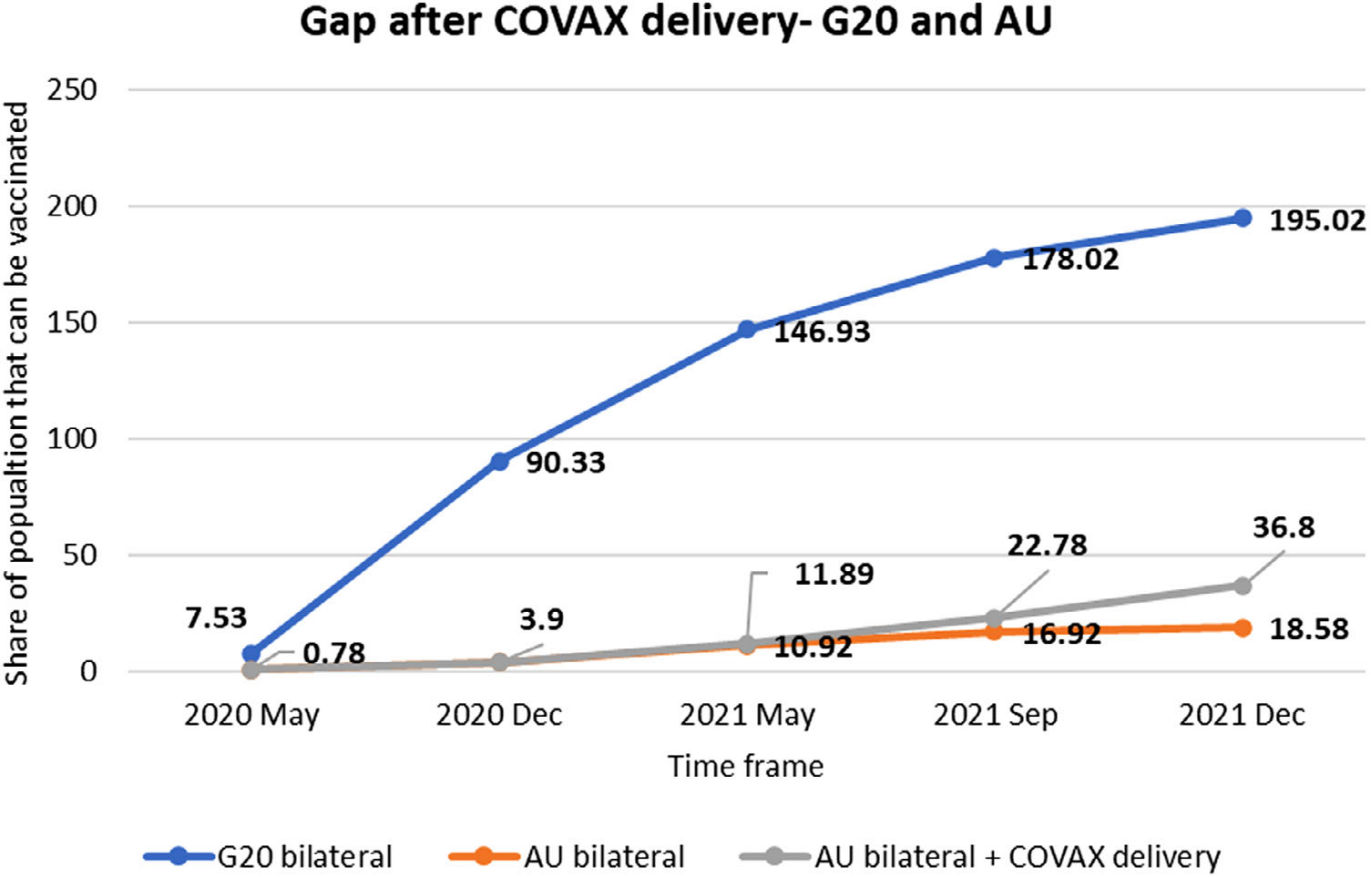
Vaccine accessibility gap after COVAX delivery—G20 versus AU, 2021 [17, 18].The data was taken from publicly reported vaccine purchase agreements by country/region mapped by the Launch and Scale Speedometer of Duke University. The data from the IMF‐WHO COVID‐19 vaccine tracker was also used. The time frame selected to plot the graph is 2020 May to 2021 December since it was in 2020 May, the United Kingdom made the world's first bilateral deal, and 2021 December is the intermediate target (40%) of WHO COVID‐19 vaccination. The blue and orange lines represent Group 20 (G20) and African Union (AU), respectively. The data on COVAX delivery for all the available countries of AU was taken from the UNICEF Vaccine market dashboard, and the cumulative figure is the bilateral purchase data.

The bilateral purchase data from Duke University Launch and Scale speedometer [16] shows that by December 2020, the G20 countries had vaccines to cover 90% of their population. After one year, that is, by December 2021, the G20 had procured enough vaccines to vaccinate double their population, whereas the AU could procure only about one fifth (19%) of their population. The five HICs that have procured the highest number of vaccines were South Korea, the USA, Japan, the United Kingdom, and Canada. All these countries independently purchased more vaccines than the entire AU. However, out of the 52 AU countries with available data, only 21 African countries could strike a bilateral deal with vaccine manufacturers. In this context, September 2021 and December 2021 denoted two important intermediate targets of the WHO global COVID‐19 vaccination strategy. According to WHO, by September 2021, the whole world should have achieved a 10% vaccination rate; by December 2021, the vaccination rate must have been 40% [7]. From the vaccine delivery data of COVAX since its rollout [17] as well as the UNICEF vaccine dashboard [18], it was apparent (Figure 2) that even after COVAX delivery, the cumulative share of the population that could be vaccinated was just 36.8% for AU, which was less than the 40% for December 2021 target.

The share of COVAX delivery was almost equal to the bilateral deals, that is, 18%. The bilateral purchase (accessibility) gap was so vast that if the vaccines were left entirely to open market dynamics, the LICs would have been left out of the vaccination. Moreover, as the G20 countries have acquired vaccines for more than 100% of their population, this essentially constitutes vaccine hoarding. For example, a single HIC in G20, Canada, has purchased COVID‐19 vaccines 4.5 times its population from the open market by the end of 2021. By then, approximately 80% of the world's total COVID‐19 vaccines were in wealthy countries, with just 0.3% of people in LICs as of April 2021 [19].

It is also important to stress that the share of vaccines delivered to AU is not similar across all the African countries. There were substantial differences between AU countries concerning the accessibility and absorbing capacity of COVID‐19 vaccines. Therefore, it was necessary to slice down the AU block further.

**FIGURE 3:**
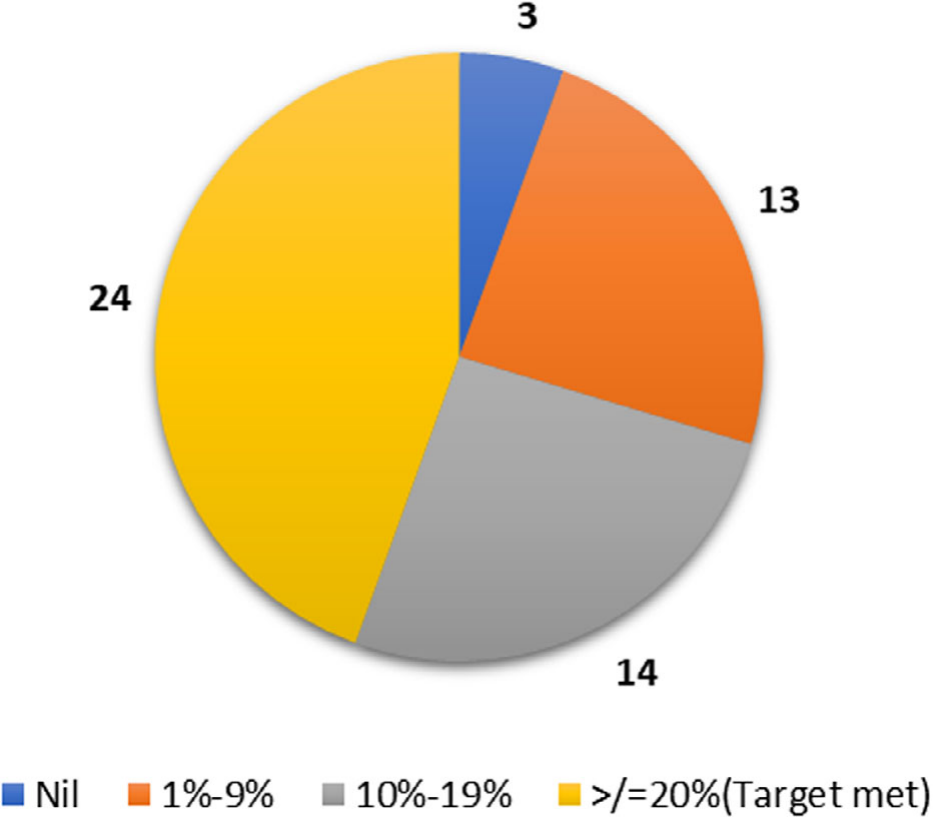
Number of African Union countries and share of population covered by COVAX delivery by December 2021 [18]. The COVAX delivery data (UNICEF vaccine market dashboard) of the African Union countries was further sliced according to the share of the population that COVAX can cover. The pie chart gives four categories of countries: the countries that met the 20% target (orange‐13), countries that can cover between 10% and 19% (gray‐14), countries that can only cover 1%–9% of the population (yellow‐24), and the countries that did not receive any vaccines from COVAX (blue‐3).

Figure 3 illustrates the share of the population exclusively covered by COVAX delivery. It was found that only 24 out of 54, that is, less than half of the AU countries (44%), met the COVAX target of covering 20% of the population by December 2021. A vast majority of the countries, mainly the LIC countries of AU, received vaccines only between 1% and 19%.

The total COVAX delivery was further assessed for those AU countries without bilateral deals, that is, those countries entirely dependent on COVAX without enough purchasing power to buy their vaccines. It was found that only nine out of 31 entirely COVAX‐dependent AU countries have received enough doses to vaccinate 20% of their population. Most LICs have received vaccines to cover 1%–19% of their population. The secondary data showed that COVAX provided vaccines to AU countries better than open market access, but not every AU country could achieve a 20% coverage target. The constraints of COVAX hindered vaccine supplies to AU countries [20]. The rampant vaccine nationalism by HICs and intellectual property and patents for COVID‐19 vaccines by pharma giants choked the supply of vaccines to this alliance [21]. The LMICs and LICs were the unfortunate sufferers of this supply constraint to COVAX, which delayed the vaccine allocation and delivery to these countries.

### Vaccine affordability

According to the UNDP Global Vaccine Equity Dashboard [6], the purchase price of COVID‐19 vaccines puts a significant financial burden on LMICs. The affordability factor of G20 was estimated using data from the UNDP COVID‐19 Vaccine Equity Dashboard and current health expenditures according to WB data [22].

The UNDP Global Vaccine Equity Dashboard data was analyzed (Table 1), and the cost of vaccinating 20% of the population (WHO target by December 2021) was merely 0.7% of the current health expenditure for G20 countries. In contrast, AU countries had to spend 5.5% of current health expenditure. The World Bank data on health expenditure as a percentage of GDP depicts that the AU already spent way less than the G20 countries due to resource constraints. Table 1 shows that to vaccinate 20% of the population, the AU countries had to spend more of their GDP than the G20 countries.

**TABLE 1:** Vaccine affordability difference between Group 20 and African Union countries [6, 22].

	Cost of vaccinating 20% of the population (a share of current health expenditure)	Current health expenditure (% of GDP)	Cost of vaccinating 20% (as a share of GDP)
G20	0.7	8.24	0.057
AU	5.5	5.20	0.291

**FIGURE 4:**
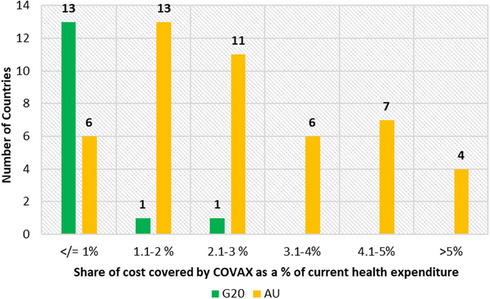
Share of health expenditure COVAX bears for G20 and AU countries, 2021 [6].The vaccine affordability data for each Group 20 (G20) and African Union (AU) country was taken from the UNDP Global Vaccine Equity Dashboard. A comparative bar graph represents the number of countries (*y*‐axis) and the corresponding share of the cost covered by COVAX (*x*‐axis).

As shown in Figure 4, COVAX bears (assuming that COVAX covers 50% of the cost of vaccines) only negligible cost of vaccinating for most G20 countries, which is less than 1.0% of expenditure. However, for most AU countries, the shared expenditure is between 1.1% and 3.0%. Thus, COVAX reduced the financial burden of the AU countries and made COVID‐19 vaccination more affordable. This shows that there was a definite need for a mechanism like COVAX to distribute vaccines, particularly to LICs.

The accessibility of vaccines, according to national income, is absurd and not justifiable because many of the phase three trials of COVID‐19 vaccines were conducted in some less developed countries. Multiple clinical trials were conducted in Africa, such as by Oxford University in Kenya and Novavax in South Africa, and the AU countries did not receive adequately developed COVID‐19 vaccines [23]. The AU countries had poor vaccine deployment infrastructure, making the vaccination more costly. Above 50% of the population in Africa lives in rural areas where the infrastructure to store and deploy the vaccines is severely limited. For example, 58% of immunization centers in Nigeria lacked electricity [24]. To combat this, in partnership with UNICEF, COVAX provided UCC (ultracold chain) facilities to most AU countries.

One of the significant causes of COVID‐19 vaccine inequity is the manufacturing capacity in LMICs that limits vaccine development and production in the Global South. As for Africa, only 1% of the vaccines needed for various infectious diseases were manufactured there [25]. The limited existing global COVID‐19 vaccine production was predominantly concentrated in Global North. The HICs prefer bilateral deals with manufacturers on their soil and invest heavily in these preferred pharma companies. In turn, these pharma companies preferred such HICs for delivering vaccines [26], and LMICs and LICs were left out of the vaccine manufacturing and technology‐transferring process. It was expected that they would have to wait longer (until late 2022) to receive vaccines as there were no binding international agreements to enforce international cooperation by capping the bilateral deals to prevent vaccine hoarding and specify the rights and obligations of countries in the context of global public goods [27].

### Vulnerability of low‐income‐countries

From the data analysis of the allocation of vaccines by COVAX, the LICs were left behind within the AU owing to their inadequate vaccine‐absorbing capacity and vaccine deployment infrastructure. For example, the Pfizer mRNA vaccine demonstrated high effectiveness against specific variants of concern. However, it was less deployed in AU countries because it lacked UCC transportation and storage facilities [28]. By the end of 2021, COVAX was able to deliver the Pfizer vaccine to only a few LICs and AU countries, such as Benin, Sierra Leone, Uganda, Malawi, Liberia, Guinea, Rwanda, DRC, Chad, Tanzania, Burkina Faso, Madagascar, Congo, Ethiopia, and Togo. COVAX could not deliver a dose of these vaccines to three LICs of AU—Burundi, Comoros, and Equatorial Guinea until December 2021. Burundi refused to participate in COVAX until recently (September 2022), citing a lack of trust in COVID‐19 vaccines [29]. The vaccine delivery to the other two countries was delayed because of a lack of supply and preparedness to receive vaccines for administration.

## CONCLUSION

COVAX failed to meet the targets by the end of 2021, but such a cooperative mechanism is fairer than the open market competition for LMICs and LICs. COVAX shared the cost of vaccinating the population with countries and provided better access to LMICs. But even with COVAX, the LICs were disproportionally hampered in vaccine accessibility, mainly because of a lack of absorptive capacity. Global wealth and income division perfectly reflects the vaccine inequity between the G20 and the AU countries. The healthcare systems of LMICs and LICs lack adequate financial and healthcare resources to absorb COVID‐19 vaccines in bulk. Improving the manufacturing capacity of vaccines in LMICs and the African continent could be a long‐term solution for global vaccine inequity. The countries of the AU should be made self‐sufficient in vaccine production to narrow the inequality gap. Healthcare structural reforms and planning are to be implemented in LICs to make them capable of absorbing pandemic vaccines for millions of people in a shorter period.

## AUTHOR CONTRIBUTIONS

*Study conception and design*: Vijay Kumar Chattu and Prakash Narayanan. *Data collection*: Anjali Pushkaran. *Analysis and interpretation of results*: Prakash Narayanan and Anjali Pushkaran. *Draft manuscript preparation*: Anjali Pushkaran. *Draft review and corrections*: Vijay Kumar Chattu and Prakash Narayanan. All authors reviewed the results and approved the final version of the manuscript.

## CONFLICT OF INTEREST STATEMENT

All authors declare that there are no conflicts of interest.

## FUNDING INFORMATION

No funds are received to conduct this study.

## Supporting information

Supporting information

## Data Availability

Data is available in the public domain. The countries taken for analysis can be found as supplementary material.
