# Supplementary material for: COVAX and COVID‐19 Vaccine Inequity: A case study of G‐20 and African Union
Source: Public Health Chall. 2024 May 9;3(2):e185. doi: 10.1002/puh2.185 (PMC12039637; doi:10.1002/puh2.185)
Supplement: Supplementary file 1 — Supporting information [file PUH2-3-e185-s001.docx]

**2.METHODS**

UNICEF and GAVI manage the data on the allocation and delivery by the COVAX in the public domain. So, using the available data sets, secondary data analysis was conducted to quantify the contribution of COVAX to bridging the gap between the High-Income countries (HICs) and Low and Middle-Income countries (LMICs) in COVID-19 vaccine equity, accessibility, and affordability. At the time of data collection (April 2022), there were 144 participants in the COVAX alliance. To capture the maximum inequity without COVAX and to estimate the contribution of COVAX, two political groups of the G20 (Group twenty) and the African Union (AU) were selected. Here (Table 1), block G20 represents the HICs with the maximum purchasing power and accessibility to COVID-19 vaccines, and AU represents the Low-Income countries (LICs) and LMICs that lack the political and purchasing power to access the vaccines. The comparative analysis between these blocks will help estimate the contribution of COVAX to LMICs and LICs, which have otherwise limited or no option for COVID-19 vaccines.

***Table 1: Number of countries in G20 and AU according to World Bank income classification^(14)^***

| **Blocks** | **Number of HICs** | **Number of UMICs** | **Number of LMICs** | **Number of LICs** | **Total** |
| --- | --- | --- | --- | --- | --- |
| G20 | 7+EU | 7 | 1 | 0 | 16 |
| AU | 1 | 6 | 21 | 24 | 52 |

The G20, the group of 20, is an international forum for economic cooperation that brings together the world's major economies representing more than 80 percent of the world GDP, 75 percent of global trade, and 60 percent of the planet's population **^(7)^**. The members of the G20 are Argentina, Australia, Brazil, Canada, China, France, Germany, India, Indonesia, Italy, Japan, the Republic of Korea, Mexico, Russia, Saudi Arabia, South Africa, Turkey, the United Kingdom, the United States, and the European Union. For the analysis, the data of France, Germany, and Italy were not taken separately, and only that of the European Union was included. Furthermore, reliable data for Russia was unavailable. So, a total of 15 countries and the European Union were included in the G20 block for data analysis. The HICs of the G20 are Canada, the USA, Japan, South Korea, Australia, Saudi Arabia, The United Kingdom, and the European Union. The UMICs of the G20 are Brazil, Mexico, Indonesia, Argentina, Turkey, South Africa, and China. India is the only LMIC in G20, and the block has no LICs.

The African Union (AU) has 55 member states from the continent of Africa **^(8)^** . Data on 53 countries of the AU were available for analysis. There was no data for Eritrea and Western Sahara, and South Africa was already included in the G20 block. So, a total of 52 countries were included in the AU block. Seychelles is the only HIC in the AU block. There are 6 UMICs in the AU block- Gabon, Namibia, Botswana, Equatorial Guinea, Mauritius, and Libya. There are 21 LMICs- Cameroon, Mauritiana, Sudan, Zambia, Nigeria, Djibouti, Lesotho, Kenya, Angola, Egypt, Senegal, Ghana, Eswatini, Zimbabwe, Comoros, Sao tome and Principe, Cape Verde, Morocco, Algeria, Tunisia and Ivory Coast. There are 24 LICs in the AU block- South Sudan, Benin, Mali, Niger, Somalia, Sierra Leone, Mozambique, Guinea Bissau, Uganda, Gambia, Liberia, Malawi, Guinea, Rwanda, DRC (Democratic Republic of Congo), Chad, Tanzania, Burkina Faso, Madagascar, CAR (Central African Republic), Congo, Burundi, Ethiopia, Togo.

The secondary data on COVAX delivery, allocation, and affordability indicators were taken from multiple open sources in the public domain, such as the Global Vaccine Equity Dashboard **^(6)^**, IMF-WHO COVID-19 vaccine tracker **^(9)^**, COVID-19 Vaccine market dashboard **^(10)^**, COVAX Rollout **^(11)^**, COVID-19 Launch and Scale Speedometer for Vaccine Equity **^(12)^** Our World in data **^(13)^** and data from World Bank **^(14)^**.
